# Preparation of High-Performance Polyethylene Nanocomposites with Oleic Acid–Siloxene-Supported Ziegler–Natta Catalysts

**DOI:** 10.3390/molecules29153662

**Published:** 2024-08-02

**Authors:** Huan Yue, Xin Yan, Chenghan Huang, Hexin Zhang, Jianming Yang, Liang Fang, Hee-Seon Kim

**Affiliations:** 1 School of Chemical & Environmental Engineering, Pingdingshan University, Pingdingshan 467000, China; 2 School of Chemistry and Chemical Engineering, Anhui University of Technology, Ma’anshan 243032, China; happysci@163.com (X.Y.); phantasm2024@163.com (C.H.); hxzhang@ahut.edu.cn (H.Z.); jianming1072@163.com (J.Y.); 3 Key Laboratory of Rubber-Plastics, Qingdao University of Science & Technology, Qingdao 266061, China; 4 Corporation R&D Center, Intelligent Construction Automation System, 80 Daehak-ro, Buk-gu, Daegu 14566, Republic of Korea; momm1225@naver.com

**Keywords:** siloxene, oleic acid, nanocomposites, mechanical properties

## Abstract

The addition of two-dimensional inorganic nanomaterials can effectively enhance the properties of polyethylene (PE). In the present study, a series of high-performance PE/oleic acid (OA)–siloxene nanocomposites were prepared by in situ polymerization using OA–siloxene-supported Ziegler–Natta catalysts. Compared with the conventional Ziegler–Natta catalyst, the polymerization activity of the OA–siloxene-supported Ziegler–Natta catalyst was enhanced to 100 kg/mol-Ti•h, an increase of 56%. The OA–siloxene fillers exhibited excellent dispersion within the PE matrix through the in situ polymerization technique. Compared to pure PE, PE/OA–siloxene nanocomposites containing 1.13 wt% content of OA–siloxene showed 68.3 °C, 126%, 37%, and 46% enhancements in *T_dmax_*, breaking strength, modulus, and elongation at break, respectively.

## 1. Introduction

Polyolefins are widely used in human life and industrial manufacturing due to their excellent properties, including easy processing, chemical stability, non-toxicity, and low cost [1,2,3,4,5]. However, the range of applications for polyolefins is largely limited by drawbacks such as low strength and poor heat resistance. Polymer nanocomposites, consisting of a continuous-phase polymer matrix and nanoparticles as fillers, have significant potential for applications. The addition of small amounts of nanofillers and their uniform dispersion throughout the polymer can significantly enhance its properties. Meanwhile, the combination of nanofillers with specific functionalities in polymers can also provide the original polymers with multifunctionality, thus expanding their areas of application. Among many nanomaterials, two-dimensional nanomaterials have received attention for their unique physical and chemical properties and have been extensively studied in the scientific literature [6,7,8,9].

Wang et al. successfully prepared polypropylene (PP)/graphene polymer composites by melt spinning and other methods. The composites with only 0.062 wt% graphene content exhibited an overall improvement in properties, with the tensile strength, modulus, and interfacial strength increased by 117%, 116%, and 116%, respectively. This improvement can be attributed to the in-plane mechanical strength and self-reinforcing mechanism of graphene [10]. Similarly, Li et al. reported that high-density polyethylene (HDPE)/graphene oxide (GO) nanocomposites containing 0.2 wt% of functionalized GO exhibited a 28.7% improvement in stress at break and a 130% improvement in strain at break [11]. Meanwhile, graphene exhibits excellent thermal stability and barrier properties, which can improve the heat resistance of composites [12]. MXene also demonstrates great potential for application in nanocomposites. Shi et al. prepared PP/MXene nanocomposites using solution casting and melt blending methods. The prepared nanocomposites exhibited significantly enhanced properties, such as an increase in the initial degradation temperature by 79.1 °C, tensile strength by 35.3%, ductility by 674.6%, and storage modulus by 102.2%. The improvement of the nanocomposites is attributed to a combination of hydrogen bonding-induced nanoconfined structures and the physical interaction of MXene nanosheets [13]. In our previous work [14,15,16,17,18], we reported a large number of polyolefin nanocomposites that significantly enhance thermal resistance and mechanical properties by utilizing only a small amount of two-dimensional fillers. This provides an effective way to produce high-performance polyolefins.

Siloxene, composed of planar geometries of silicon and oxygen, has recently emerged as a promising two-dimensional material with potential applications beyond MXene and graphene [19]. Siloxene is obtained by removing Ca from calcium disilicide (CaSi_2_) and has a two-dimensional corrugated structure consisting of Si_6_ rings [20]. Siloxene maintains a stoichiometric ratio of Si:H:O as 2:2:1. Research has demonstrated the existence of two distinct structures of siloxene, namely, the Weiss structure and the Kautsky structure, depending on the exfoliation and deposition conditions, which include reaction time, the concentration of the acidic medium, and temperature [21]. In Weiss-type siloxenes (Si_6_(OH)_3_H_3_), the six-membered Si_6_ ring is connected with alternating substituted Si-H and Si-OH bonds, whereas in Kautsky-type siloxenes (Si_6_O_3_H_6_), the Si_6_ ring is connected with Si-O-Si bridges and hydroxyl functional groups exist on the surface [22,23,24]. Siloxene is distinguished from graphene and graphene oxide by its unique two-dimensional, low-buckled structure, which provides good flexibility and an amorphous structure [20,21]. Previous research has focused on the potential applications of siloxenes in the fields of catalysis, capacitor electrodes, and semiconductors. The unique two-dimensional structure and the abundance of functional groups endow siloxene with a variety of advantages. However, research on the application of siloxenes as polymer-modified fillers in nanocomposites is very scarce.

In this study, we synthesized an oleic acid (OA)–siloxene-supported Ziegler–Natta catalyst and evaluated the catalytic activity in ethylene polymerization in the presence of triethylaluminum (TEA) as a co-catalyst. The catalyst exhibited excellent activity towards ethylene, and a homogeneous dispersion of OA–siloxene fillers in the polyethylene (PE) matrix was obtained after in situ polymerization. Compared to pure PE, the PE/OA–siloxene nanocomposites with minimal OA–siloxene fillers demonstrated superior heat resistance and mechanical properties.

## 2. Results and Discussion

**Scheme 1:**
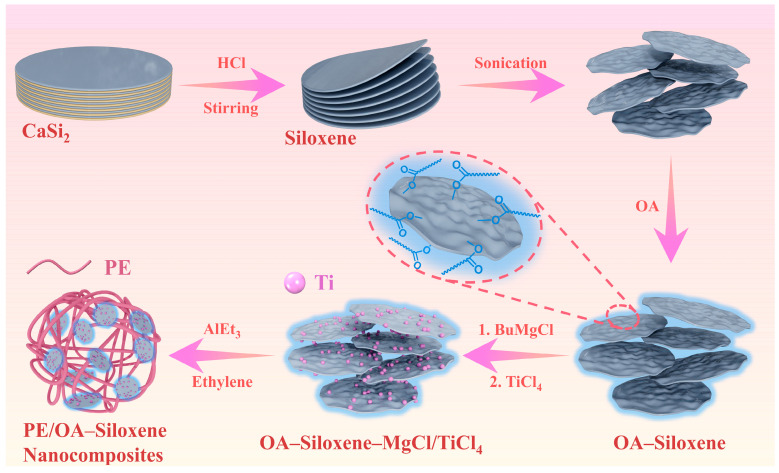
Preparation of siloxene, OA–siloxene, OA–siloxene–MgCl/TiCl_4_ catalyst, and PE/OA–siloxene nanocomposites.

The preparation of siloxene, OA–siloxene, OA–siloxene–MgCl/TiCl_4_ catalyst, and PE/OA–siloxene nanocomposites via in situ polymerization is illustrated in Scheme 1. Siloxene nanosheets were obtained by treating CaSi_2_ with concentrated HCl to remove Ca atoms and ultrasonically exfoliate the resulting solid. The siloxene nanosheets were modified with OA and then dispersed in a dry THF solution. Excess BuMgCl was added under N_2_. The reaction process mainly involves the reaction of BuMgCl with the -OH groups in the OA–siloxene filler, followed by the anchoring of TiCl_4_ to obtain the OA–siloxene–MgCl/TiCl_4_ catalyst. Following TEA co-catalyst activation, the OA–siloxene–MgCl/TiCl_4_ catalysts were evaluated in ethylene polymerization, and a series of high-performance PE/OA–siloxene nanocomposites were successfully fabricated.

**Figure 1:**
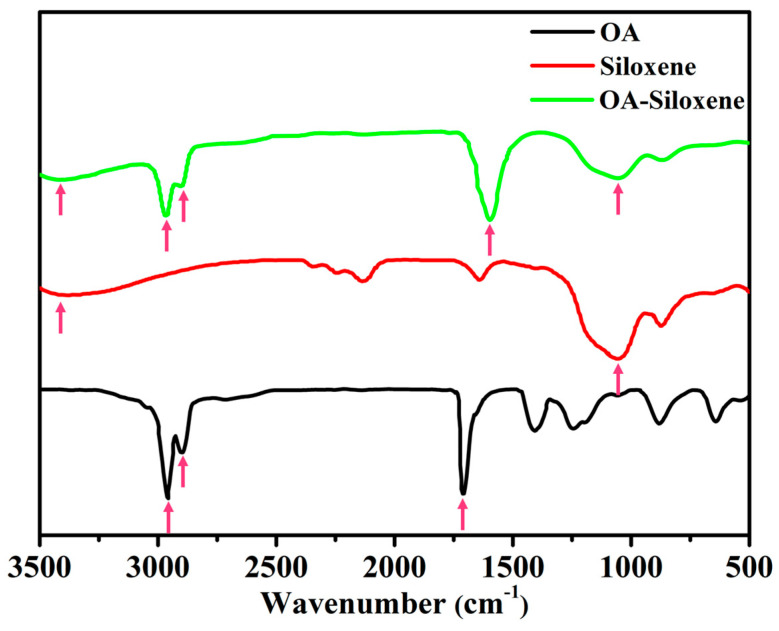
FTIR spectra of OA, siloxene, and OA–siloxene.

To determine the success of the modification, the chemical structures of siloxene, OA, and OA–siloxene were characterized using FITR (Figure 1). In the FTIR of siloxene, the absorption peak at 3388 cm^−1^ corresponds to the hydroxyl functional groups existing on siloxene, and the absorption peak at 1056 cm^−1^ corresponds to the Si-O-Si vibration [22,25]. As given in Figure 1, the absorption bands at 2955 and 2899 cm^−1^ were observed in both OA and OA–siloxene nanosheets, which are the characteristic signals of -CH_2_- of OA [26], indicating the presence of a long alkyl chain in OA–siloxene. The -COOH functional group in OA is indicated by the peak absorption at 1711 cm^−1^. However, no significant absorption peak was observed for OA–siloxene at 1711 cm^−1^, but instead a new absorption peak corresponding to the -COO- stretching vibration appeared at 1597 cm^−1^, suggesting the formation of a covalent bond between OA and siloxene [27,28]. Therefore, OA was effectively grafted onto the siloxene surface.

**Figure 2:**
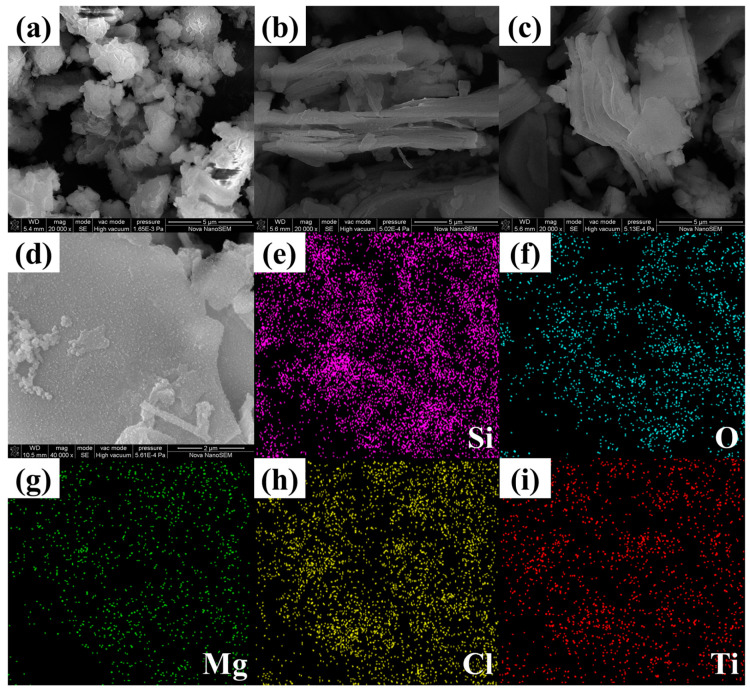
SEM images of (**a**) CaSi_2_, (**b**) siloxene, (**c**) OA–siloxene, and (**d**) OA–siloxene–MgCl/TiCl_4_ catalyst at different magnifications, and (**e**–**i**) EDX elemental mapping of Si, O, Mg, Cl, and Ti from (**d**).

The micromorphology of CaSi_2_, siloxene, OA–siloxane, and OA–siloxene–MgCl/TiCl_4_ catalysts was investigated using SEM. The SEM image in Figure 2a shows that the CaSi_2_ sample exhibits a blocky structure with a diameter of about a few micrometers. The siloxene nanosheets obtained after treatment with concentrated HCl exhibited a layered structure with some spacing. This phenomenon can be attributed to the removal of the Ca layer in the CaSi_2_ powder using selective etching with concentrated HCl. Compared to siloxene, there were no significant changes in the microscopic morphology of OA–siloxene, and the two-dimensional layered structure remained. The lamellar structure of the OA–siloxene–MgCl/TiCl_4_ catalyst exhibited numerous dots on the surface, which can be attributed to the anchoring of TiCl_4_. Meanwhile, the homogeneous distribution of the dot structure indicates that the Ti metal activity is uniformly dispersed on the siloxene nanosheets. To obtain more clear evidence, EDX analysis was performed to characterize the elemental distribution in the OA–siloxene–MgCl/TiCl_4_ catalysts. Figure 2e–i shows the EDX elemental mapping images of Si, O, Mg, Cl, and Ti. The images showed that the Ti active sites were uniformly distributed throughout the OA–siloxene fillers. The Ti content of the OA–siloxene–MgCl/TiCl_4_ catalyst was 7.3 wt%, while the Ti content of the MgCl_2_/TiCl_4_ catalyst was 7.1 wt%, as determined by inductively coupled plasma atomic emission spectroscopy (Table 1).

**Figure 3:**
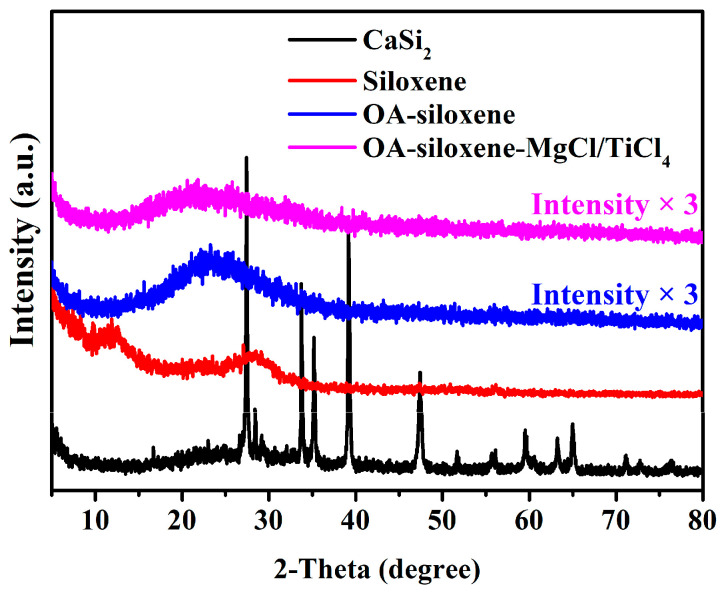
XRD pattern of CaSi_2_, siloxene, OA–siloxene, and OA–siloxene–MgCl/TiCl_4_ catalyst.

The CaSi_2_, siloxene, OA–siloxene, and OA–siloxene–MgCl/TiCl_4_ catalyst were also investigated using XRD. After CaSi_2_ was etched using concentrated HCl, the microstructure changed significantly. As shown in Figure 3, the sharp, narrow diffraction peaks in the XRD peaks of CaSi_2_ indicate that it is highly crystalline. However, the XRD spectrum of siloxene demonstrated that the peaks corresponding to the characteristic lattice planes of CaSi_2_ disappeared, and the peaks became broad and weak, indicating that the Ca atoms had been removed from CaSi_2_ [13]. Excitingly, the peak at 2θ = 11.92° (corresponding to a layer spacing of 7.42 nm) disappears for OA–siloxene compared to the peak for siloxene, and the peak at 2θ = 23.26° is still very weak and broad despite a 3-fold amplification of the intensity, suggesting that most of the stacked siloxene layers can be efficiently intercalated and exfoliated by grafting with OA. Notably, the diffraction peaks of the OA–siloxene–MgCl/TiCl_4_ catalyst at 2θ = 23.26° became weaker and broader, proving that the catalyst was uniformly distributed and successfully anchored to the surface of the siloxene nanosheets.

The catalytic activity of the MgCl_2_/TiCl_4_ and OA–siloxene–MgCl/TiCl_4_ catalysts in ethylene polymerization was evaluated with triethanolamine (TEA) as a co-catalyst. As summarized in Table 2, the activity of OA–siloxene–MgCl/TiCl_4_ catalysts exhibits an increasing trend with the increase in the [Al]/[Ti] ratio at the same amount of catalyst addition (Entry 2~6). At an [Al]/[Ti] ratio of 90, the catalytic activity of the MgCl_2_/TiCl_4_ catalyst was 64 kg/mol-Ti•h (Entry 1), whereas the ethylene polymerization activity of the OA–siloxene–MgCl/TiCl_4_ catalyst at the same catalyst feed was 100 kg/mol-Ti•h (Entry 10). The higher activity of the OA–siloxene–MgCl_2_/TiCl_4_ catalyst could be attributed to the uniform anchoring of catalyst active centers on the nanosheets, which effectively prevented catalyst aggregation accompanied by an increase in the surface of the catalysts. In this study, PE/OA–siloxene nanocomposites containing 0.54–1.13 wt% of OA–siloxene filler were achieved by changing the amount of catalyst feed and the [Al]/[Ti] ratio.

**Figure 4:**
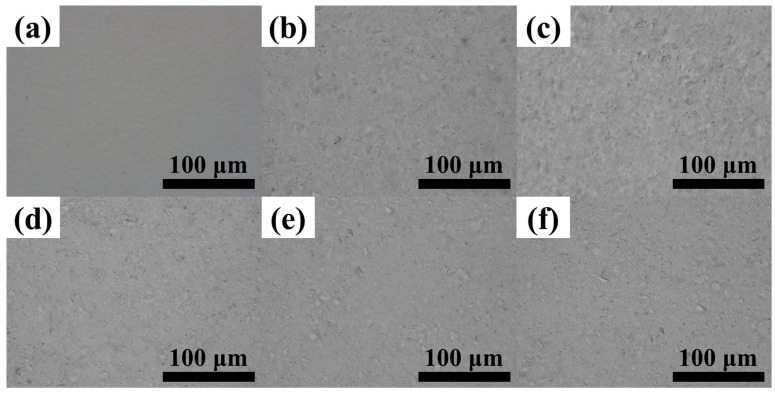
Optical micrographs of (**a**) PE and PE/OA–siloxene nanocomposites with different OA–siloxene contents: (**b**) 0.54 wt%, (**c**) 0.79 wt%, (**d**) 0.96 wt%, (**e**) 1.05 wt%, and (**f**) 1.13 wt%.

To assess the dispersion state of OA–siloxene nanosheets in the polymer, PE/OA–siloxene nanocomposites were analyzed using optical microscopy. All samples were hot-pressed into thin films. The dispersion state of OA–siloxene fillers in the polymer matrix can be clearly observed with an optical microscope, as recorded in Figure 4. From Figure 4b, it can be clearly seen that the OA–siloxene fillers are uniformly dispersed in the PE matrix. Further, as the filler content increases, more OA–siloxene fillers can be seen in the PE matrix. Excitingly, there is no obvious aggregation in the series of PE/OA–siloxene nanocomposites prepared in this study.

**Figure 5:**
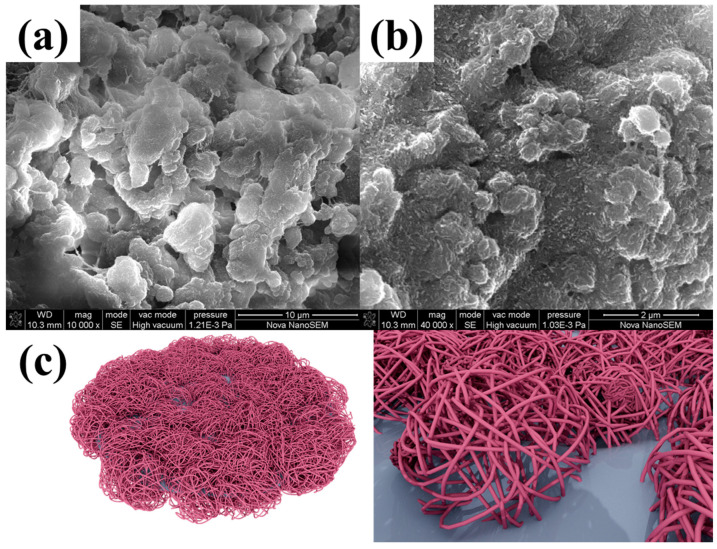
SEM images of (**a**,**b**) PE/OA–siloxene 1.13 nanocomposites at different magnifications and (**c**) the mechanism of the microscopic morphology formation of nanocomposites.

It is well known that heterogeneous catalysts have an excellent ability to control polymer morphology. Therefore, the morphology of the obtained PE/OA–siloxene nanocomposite particles was investigated. As shown in Figure 5a,b, the prepared PE/OA–siloxene nanocomposite particles showed an elliptical shape with a size of about 7 μm, with spherical particles less than 1 μm in size aggregated on the surface. Meanwhile, small villi of the polymer can be observed on the surface of the particles. The possible mechanism for PE/OA–siloxene nanocomposites to exhibit this morphology is demonstrated in Figure 5c. A large amount of Ti metal active centers was loaded on the surface of the OA–siloxene nanosheets. And the polymerization of the ethylene monomer was initiated by the activation of TEA. During the polymerization process, PE formed and aggregated into spherical particles on the siloxene nanosheet layer. Tiny villi may arise due to the extrusion of the polymer from the bulk of large particles to the surface. Meanwhile, no OA–siloxene nanosheets were clearly observed from the surface of PE/OA–siloxene nanocomposite particles, which proved that the siloxene fillers were tightly wrapped in the PE.

**Figure 6:**
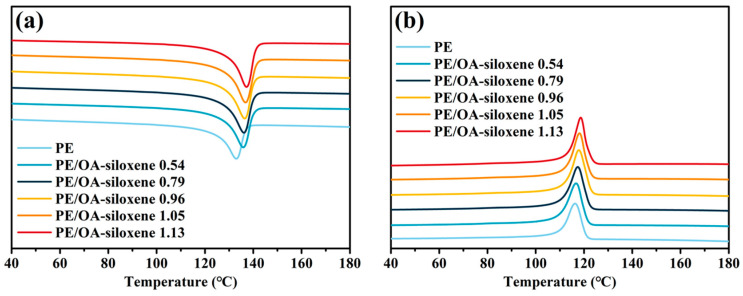
DSC (**a**) heating and (**b**) cooling curves of PE and PE/OA–siloxene nanocomposites with different OA–siloxene contents.

The impact of OA–siloxene fillers on the melting temperature (*T_m_*) and crystallization temperature (*T_c_*) of PE/OA–siloxene nanocomposites was analyzed using DSC, as outlined in Table S1 and Figure 6. The *T_m_* of pure PE prepared with MgCl_2_/TiCl_4_ catalyst was 133.0 °C. For PE/OA–siloxene nanocomposites, the *T_m_* was elevated up to 137.2 °C with the increase in the content of OA–siloxene fillers. This enhancement can be attributed to the interaction between the OA–siloxene fillers and the PE matrix, which limits the mobility of the PE macromolecular chains. Moreover, the *T_c_* and crystallinity degree (*X_c_*) of PE/OA–siloxene nanocomposites also increased with the increase in the content of OA–siloxene fillers. Specifically, the *T_c_* and *X_c_* of pure PE were 116.5 °C and 46.3%, respectively. Compared to pure PE, the *T_c_* and *X_c_* of PE/OA–siloxene 1.13 nanocomposites increased to 118.7 °C and 60.4%. This suggests that the OA–siloxene fillers act as a nucleating agent (heterogeneous nucleation) and promote the crystallization of PE. It is no coincidence that inorganic fillers have a good nucleating effect on polyolefins, which has been widely proven [29,30,31]. Meanwhile, the DSC curves of all the samples showed smooth profiles with sharp endothermic peaks, which also indicated that the OA–siloxene fillers were uniformly distributed in the PE matrix and no aggregation occurred.

**Figure 7:**
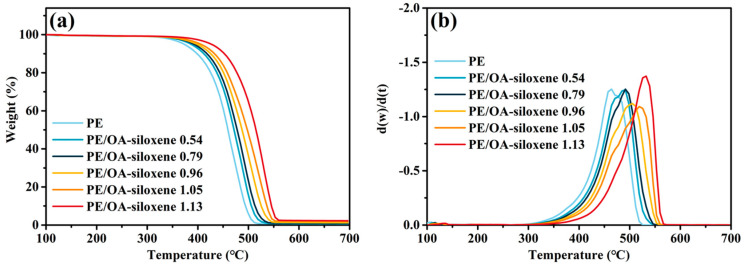
(**a**) TGA and (**b**) DTG curves of PE and PE/OA–siloxene nanocomposites.

Thermal stability is one of the most critical properties of polyolefin materials as it affects the processability and service life of these materials. Therefore, we evaluated the thermal stability of pure PE and PE/OA–siloxene nanocomposites using TGA, which is summarized in Table S2 and Figure 7. It is clear to see that the TGA curves of both PE and PE/OA–siloxene nanocomposites exhibit a single degradation step. Compared with pure PE, the thermal degradation curves of PE/OA–siloxene nanocomposites shifted to high temperatures with the increase in OA–siloxene content, which proved that the OA–siloxene fillers effectively enhanced the thermal stability of PE. As shown in Table S2, the *T_d5%_* and *T_dmax_* of all PE/OA–siloxene nanocomposites were higher than those of pure PE. Specifically, the *T_d5%_* and *T_dmax_* of PE/OA–siloxene nanocomposites increased by 53.8 and 68.3 °C, respectively. The significant elevation in thermal stability of PE/OA–siloxene nanocomposites with the introduction of siloxene nanosheets is attributed to the excellent thermal stability of the two-dimensional siloxene itself and its excellent dispersion in the PE matrix. The siloxene fillers act as a two-dimensional insulating barrier, isolating the heat source from the polymer surface.

In addition, at 650 °C, the carbon residue of all PE/OA–siloxene nanocomposites was improved compared to that of pure PE. As shown in Table S2, the residual carbon amounts of the nanocomposites with OA–siloxene contents of 0.54, 0.79, 0.96, 1.05, and 1.13 wt% were 0.8, 0.9, 1.2, 1.9, and 2.3 wt%, respectively. From these results, it is easy to see that siloxene has a layered structure, which promotes the carbonization of PE and improves the thermal stability of the polymers [32,33]. Layered nanofillers can enhance the thermal stability of polymers by their physical barrier effect, hindering the diffusion of degradation products, gasses, and heat.

**Figure 8:**
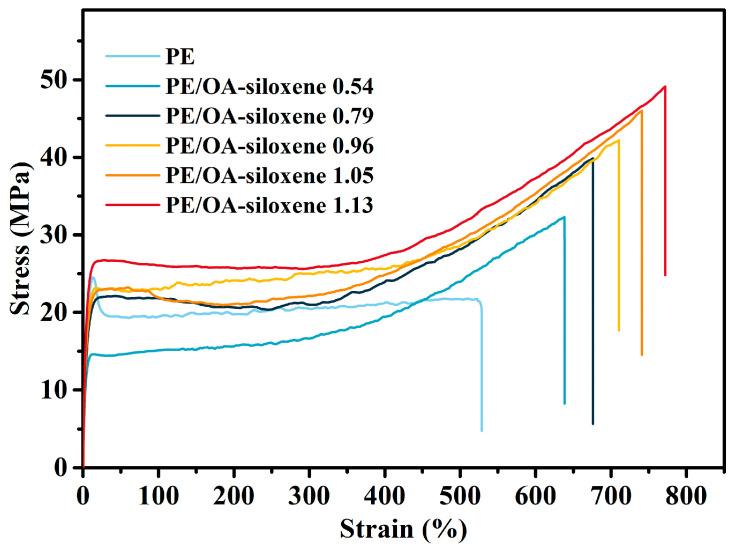
Stress–Strain behavior of PE and PE/OA–siloxene nanocomposites.

It is well established that the dispersion state and compatibility of nanofillers within the matrix significantly determine the mechanical properties of nanocomposites. Due to the presence of long-chain alkyl groups, OA–siloxene has excellent interfacial compatibility with the PE matrix. During the polymerization process, the ethylene monomer was polymerized using OA–siloxene as a template (Figure 5). The PE/OA–siloxene nanocomposites showed an excellent dispersion of OA–siloxene fillers. Moreover, the POM and XRD results revealed that the OA–siloxene was completely exfoliated and homogeneously dispersed in the PE matrix, and thus it was expected that the introduction of OA–siloxene nanosheets significantly enhanced the mechanical properties of PE. For the purpose, we evaluated the mechanical properties of PE and PE/OA–siloxene nanocomposites with different OA–siloxene contents (Figure 8 and Table S3). It is noteworthy that pure PE prepared with the MgCl_2_/TiCl_4_ catalyst did not exhibit a significant stress-hardening behavior. This phenomenon can be attributed to the lower crystallinity of PE (46.3%). The results showed that the mechanical properties of OA–siloxene nanocomposites were significantly improved even with very low contents of 0.54, 0.79, 0.96, 1.05, and 1.13 wt%, respectively. Among them, the PE/OA–siloxene nanocomposites with a content of only 1.13 wt% displayed the maximum increase in breaking strength, modulus, and elongation at break of 126%, 37%, and 46%, respectively. Hence, the mechanical properties of PE/OA–siloxene nanocomposites prepared with OA–siloxene–MgCl/TiCl_4_ catalysts were comprehensively improved compared with those of pure PE prepared with conventional MgCl/TiCl_4_.

## 3. Materials and Methods

### 3.1. Synthesis of OA–Siloxene

All the freeze-drying experiments were carried out with an FD-1A-50 freeze-dryer (Shanghai Gipp Electronic Technology Co., Ltd., Shanghai, China) at −50°C under a vacuum. The two-dimensional siloxene nanosheets were synthesized based on the literature [34]. Briefly, an appropriate amount of calcium disilicide (CaSi_2_) was reacted with concentrated hydrochloric acid (HCl) at a low temperature for at least 96 h. Throughout the reaction, Ca atoms were removed from CaSi_2,_ accompanied by a change in the color of the solid from black to yellowish. The obtained solid was washed with deionized water several times to neutralize it. The exfoliated siloxene nanosheets were obtained after freeze-drying. Subsequently, the 5 g exfoliated siloxene was dispersed in a *n*-hexane solution of OA with vigorous stirring at 60 °C for 4 h. The suspension was then filtered and washed several times with a mixture of alcohol and deionized water. Immediately thereafter, OA–siloxene powder was obtained through a freeze-drying process.

### 3.2. Preparation of OA–Siloxene–MgCl/TiCl_4_ Ziegler–Natta Catalyst

All the freeze-drying experiments were carried out with an FD-1A-50 freeze-dryer at −50 °C under vacuum. The whole preparation process was carried out in an anhydrous and oxygen-free environment. After purging the 500 mL reactor with N_2_ three times, 2 g of OA–siloxene was added. Then, 200 mL of tetrahydrofuran (THF) was fed, and the suspension was ultrasonicated for 1 h at room temperature. An excess of *n*-butylmagnesium chloride (BuMgCl) was slowly introduced to the above suspension at 0 °C. The reaction temperature was increased to 80 °C within 1 h and maintained for 4 h. The solid product was subsequently filtered under N_2_ protection and washed with THF to eliminate any unreacted BuMgCl. After removing THF under vacuum, 200 mL of hexane was introduced and dispersed by means of ultrasonication for 1 h. Finally, 20 mL of titanium tetrachloride (TiCl_4_) was slowly injected dropwise under an ice bath and stirred for 1 h at this temperature. The temperature was then increased to 80 °C, and the reaction was allowed to run for 6 h. The product was washed several times with hot *n*-hexane to remove residual TiCl_4_. Then, an OA–siloxene–MgCl/TiCl_4_ catalyst was obtained. For comparison, a catalyst without OA–siloxene (MgCl_2_/TiCl_4_) was also prepared through the reaction of MgCl_2_ with TiCl_4_.

The “materials”, “ethylene polymerization”, and “characterization” sections of the experiments are available in the “Supporting Information”.

## 4. Conclusions

In this study, an OA–siloxane-supported Ziegler–Natta catalyst was successfully prepared by reacting OA–siloxene with BuMgCl and then anchoring TiCl_4_. The polymerization results showed that the prepared OA–siloxene-supported Ziegler–Natta catalyst exhibited excellent ethylene polymerization performance (enhanced by 56%) compared to the MgCl_2_/TiCl_4_ catalyst. In addition, the fillers in the PE/OA–siloxene nanocomposites prepared by means of in situ polymerization were uniformly dispersed, which greatly enhanced the thermal stability and mechanical properties of the nanocomposites. The PE/OA–siloxene nanocomposites containing 1.13 wt% OA–siloxene fillers exhibited *T_d5%_*, breaking strength, modulus, and elongation at break enhancements of 53.8 °C, 126%, 37%, and 46%, respectively. Thus, a series of high-performance PE/OA–siloxene nanocomposites with strength, toughness, and thermal stability were developed in this study.

## Figures and Tables

**Table 1:** Elemental composition of catalysts.

Catalyst	Mg (wt%)	Ti (wt%)	[Ti]/[Mg] (mole/mole)
MgCl_2/_TiCl_4_	25.2	7.1	0.14
OA–siloxene–MgCl/TiCl_4_	5.4	7.3	0.67

**Table 2:** Results of ethylene polymerization by MgCl_2_/TiCl_4_ catalyst and OA–siloxene–MgCl/TiCl_4_ catalyst ^a^.

Entry	Cat.	Cat. (mg)	[Al]/[Ti] ^b^	Yield (g)	Activity (kg/mol-Ti•h)	OA–Siloxene (wt%)
1	MgCl_2_/TiCl_4_	100	90	4.7	64	0
2	OA–siloxene–MgCl/TiCl_4_	15	18	0.7	57	1.99
3		15	36	1.2	103	1.11
4		15	54	1.8	155	0.73
5		15	90	2.4	212	0.54
6		15	140	2.7	239	0.47
7		30	90	3.3	144	0.79
8		45	90	4.0	118	0.96
9		60	90	4.9	108	1.05
10		100	90	7.6	100	1.13

^a^ Polymerization conditions: 1 atm, 0.5 h, 40 °C, 100 mL *n*-hexane, TEA as cocatalyst. ^b^ [Al]/[Ti] molar ratio.

## Data Availability

Data will be made available upon request.

## Supplementary Materials

The following supporting information can be downloaded at: https://www.mdpi.com/article/10.3390/molecules29153662/s1, Figure S1. Digital photos showing CaSi_2_ and siloxene. Table S1. *T_m_*, *T_c_* and *X_c_* Results of PE and PE/OA–siloxene Nanocomposites. Table S2. Effect of OA–siloxene content on thermal stabilities of PE/OA–siloxene nanocomposites. Table S3. Mechanical Properties of PE and PE/OA–siloxene nanocomposites with various OA-siloxene contents [35,36,37].
