# Supplementary material for: Preparation of High-Performance Polyethylene Nanocomposites with Oleic Acid–Siloxene-Supported Ziegler–Natta Catalysts
Source: Molecules. 2024 Aug 2;29(15):3662. doi: 10.3390/molecules29153662 (PMC11314101; doi:10.3390/molecules29153662)
Supplement: Supplementary file 1 [file molecules-29-03662-s001.zip › molecules-3132080-supplementary.pdf]

Supplementary Materials

Materials

Tetrahydrofuran (THF, >99.5%), triethylaluminum (TEA, 2.0M in hexane), calcium disilicide ($CaSi_2$, >99%, 3~5 μm), *n*-butylmagnesium chloride (BuMgCl, 2.0M in THF), *n*-hexane (>97%) and titanium tetrachloride ($TiCl_4$, >99%) were sourced from Shanghai Titan Technology Co. Ltd., China. Oleic acid (OA, >99%) was purchased from Shanghai Adamas Reagent Co., Ltd. Ethylene (polymerization-grade) was provided by Nanjing Special Gas Factory Co., Ltd., China. Hydrochloric acid (HCl, 37%) was bought from Sinopharm Chemical Reagent Co. Ltd., China. THF and *n*-hexane underwent distillation over sodium/benzophenone under a nitrogen atmosphere for purification before being utilized.

Ethylene polymerization

All polymerization reactions were required to be carried out in a N_2-purged environment. Polyethylene (PE) and PE/OA-siloxene nanocomposites were prepared in a glass reactor equipped with a magnetic stirrer. After the reactor reached the reaction temperature, ethylene (1 atm) was introduced and the pressure was ensured to be constant throughout the reaction. TEA was then added, followed by the injection of catalyst to initiate the polymerization reaction. The polymerization reaction was terminated after the introduction of HCl-methanol solution. The collected polymer was dried under vacuum at 60 °C until a constant weight was achieved. The resulting products were named based on the mass fraction of OA-siloxene in the PE matrix; for

instance, nanocomposites containing XX wt% of OA-siloxene were named PE/OA-siloxene XX, while the control group was named PE.

Characterization

Inductively coupled plasma atomic emission spectroscopy (ICP-AES) analysis (ICPS-7510 PLUS) was performed to determine the elemental contents in the catalyst. SEM images were obtained with a NANO SEM430 microscope, and X-ray diffraction patterns were recorded using a D8 Advance diffractometer with Cu-Kα radiation. Optical microscopy (LW300JT) was employed to capture the morphologies of the support and catalyst.

Melting temperature (T_m) and crystallization temperature (T_c) were analyzed using differential scanning calorimetry (DSC 2500). The sample underwent heating to 200 °C, maintained for 3 minutes to eliminate thermal history effects, and was subsequently cooled to a temperature of 30 °C. T_m was recorded during the second scan. Decomposition temperature (T_d) analysis was carried out using thermogravimetric techniques (DTG-60H) under a nitrogen atmosphere within a temperature range of 20 to 700 °C. Tensile mechanical properties of PE and PE/OA-siloxene nanocomposites were evaluated using an AGS-X universal testing machine.

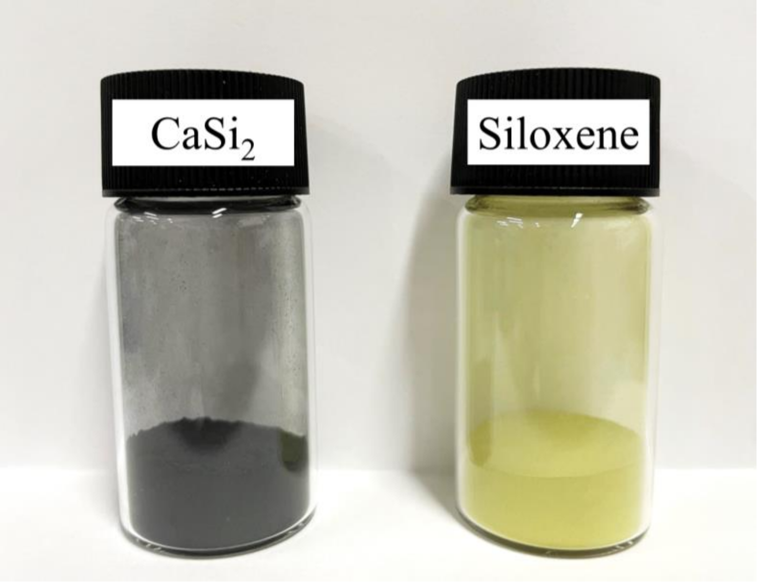

Figure S1. Digital photos showing $CaSi_2$ and siloxene.

Table S1. T_m, T_c and X_c Results of PE and PE/OA-siloxene Nanocomposites.

Entry	OA-siloxene (wt%)	T_m (°C)	T_c (°C)	ΔH_f (J/g)	X_c (%)
Neat PE	0	133.0	116.5	134.7	46.3
PE/OA-siloxene Nanocomposites	0.54	135.8	116.8	158.0	54.3
	0.79	136.1	117.5	161.2	55.4
	0.96	136.4	117.9	168.2	57.8
	1.05	136.8	118.2	172.0	59.1
	1.13	137.2	118.7	175.8	60.4

The degree of crystallinity (X_c) of all samples was calculated by the following formula equation, where ΔH_f is the enthalpy of melting of the sample and ΔH_f^* is the enthalpy of melting of 100% crystalline PE, which is 291 (J/g) [35-37].

Table S2. Effect of OA-siloxene content on thermal stabilities of PE/OA-siloxene nanocomposites.

Entry	OA-siloxene (wt%)	$T_{d5\%}$ (°C)	T_{dmax} (°C)	Char yield (wt%)
Neat PE	0	372.9	464.2	0.7
PE/OA-siloxene Nanocomposites	0.54	386.7	491.5	0.8
	0.79	392.3	491.5	0.9
	0.96	398.4	505.2	1.2
	1.05	405.7	518.8	1.9
	1.13	426.7	532.5	2.3

Table S3. Mechanical Properties of PE and PE/OA-siloxene nanocomposites with various OA-siloxene contents.

	OA-siloxene (wt%)	Breaking Strength (MPa)	Modulus (MPa)	Elongation at Break (%)
Neat PE	0	21.7±1	482±6	528±15
PE/OA-siloxene Nanocomposites	0.54	32.3±2	561±7	638±10
	0.79	39.9±2	572±8	676±12
	0.96	42.2±3	577±6	711±15
	1.05	46.0±1	615±5	741±13
	1.13	49.1±3	662±6	772±13
